# IGF2BP1, a New Target to Overcome Drug Resistance in Melanoma?

**DOI:** 10.3389/fphar.2022.947363

**Published:** 2022-07-22

**Authors:** Yufeng Xi, Yujia Wang

**Affiliations:** ^1^ Department of Neonatology, Chengdu Women’s and Children’s Central Hospital, School of Medicine, University of Electronic Science and Technology of China, Chengdu, China; ^2^ Department of Dermatology, State Key Laboratory of Biotherapy, West China Hospital, Sichuan University, Chengdu, China

**Keywords:** IGF2BP1, drug resistance, melanoma, RNA binding protein, mRNA stabilization

Insulin like growth factor 2 mRNA binding protein 1 (IGF2BP1) belongs to a conserved RNA binding protein family, including IGF2BP1, IGF2BP2, and IGF2BP3. IGF2BPs contain six important domains, including two N-terminal RNA recognition binding domains and four C-terminal ribonucleoprotein-K homology. This structural feature allows IGF2BPs to bind to a variety of target mRNAs and then regulate the expression level of target genes at the post-transcriptional level (Wächter et al., 2013). IGF2BP1 is highly expressed in a variety of malignant tumors, and it is inclined to be considered as an oncogene. IGF2BP1 is involved in the proliferation, adhesion, and migration in tumor development (Bell et al., 2013). Since Elcheva et al. (2008) first reported the role of IGF2BP1 in melanoma in 2008, we note that IGF2BP1 plays an important role in melanoma resistance and hereby summarize the latest research on it.

It has been reported that IGF2BP1 may serve as a prognostic marker in melanoma, on account of IGF2BP1 being overexpressed in metastatic melanoma, which leads to resistance to chemotherapeutic agents (Craig and Spiegelman, 2012; Fortis et al., 2017). Although BRAF inhibitors significantly improve survival in patients with metastatic melanoma, most patients relapse less than a year after treatment (Robert et al., 2015). Inhibition of IGF2BP1 can enhance the role of BRAF inhibitors and BRAF-MEK inhibitors in BRAFV600E melanoma. Besides, knockout of IGF2BP1 alone is sufficient to reduce the tumorigenicity of vemurafenib-resistant melanoma (Kim et al., 2018). Archita Ghoshal et al. reported that the RNA-binding protein IGF2BP1 played a critical role in melanoma metastasis. The suppression of IGF2BP1 did not inhibit primary tumor formation, but lung metastasis has been significantly inhibited. It has been proposed for the first time that RNA-binding protein played a role in EV-mediated promotion of metastasis. EVs (extracellular vesicles) from IGF2BP1-overexpressed melanoma cells further accelerated EV-induced metastasis. And EVs from IGF2BP1 knockdown melanoma cells affected the metastasis microenvironment *via* inhibiting fibronectin deposition and CD45^+^ cell accumulation in the lung; thus, EVs from IGF2BP1 knockdown melanoma cells cannot promote metastasis. IGF2BP1 was closely related to the regulation of the cargo of EVs, thus affecting the pro-metastatic function of melanoma-derived EVs, which may open a new way for the development of potential inhibitors for cancer treatment (Ghoshal et al., 2019). In addition, the conservative interaction between THOR and IGF2BP1 has been reported, and it has been shown that THOR was conducive to the mRNA stabilization activities of IGF2BP1 in the zebrafish model of melanoma (Hosono et al., 2017). p62 interacted with IGF2BP1 *via* the PB1 domain in UACC-62 cells (melanoma cell lines) and then controlled the mRNA stability of FERMT2 and multiple pro-metastatic factors (EHMT2, CD2AP, TOP2A, FLOT1, OGFOD1, and NCEH1) (Karras et al., 2019). Inhibition of IGF2BP1 can reduce the stability of PKCα mRNA, the expression of PKCα protein, and MAPK/ERK activation to improve overall survival in melanoma (Mahapatra et al., 2019). As for IGF2BP1 inhibitor, BTYNB could widely inhibit the activity of IGF2BP1 in a variety of tumors. It disrupts IGF2PB1-RNA binding to inhibit post-transcriptional “super”-enhancer action in E2F-driven gene expression (Müller et al., 2020). When combined with several common chemotherapeutic drugs and CDK inhibitors, BTYNB has a synergistic effect on the inhibition of the proliferation of neuroblastoma cells (Biegel et al., 2021). At present, chemotherapy drugs are widely used in clinical practice and CDK inhibitors are also approved for marketing, IGF2BP1 inhibitors combined with existing clinical drugs may lead to higher efficiency and lower drug resistance and toxicity, thus bringing better therapeutic effects for patients. Another IGF2BP1 inhibitor termed “7773” can directly bind IGF2BP1 and inhibit its binding to Kras RNA (Wallis et al., 2022). Kras is a common mutation site in tumors, which is difficult to target. So far, no Kras target drug has been approved for clinical use. Therefore, the IGF2BP1 inhibitor “7773” has potential clinical value. In the treatment of melanoma, BTYNB was identified as an effective and selective inhibitor of IGF2BP1 binding to c-myc mRNA to reduce melanoma cell proliferation (Mahapatra et al., 2017). IGF2BP1 stabilized MITF mRNA and increased its expression and its transcriptional activity, which was mediated by counteracting the miR-340–mediated degradation of MITF mRNA (Goswami et al., 2015). Many studies have shown that IGF2BP1 inhibitors have potential clinical application, whether used alone or in combination with other drugs.

Those findings suggested that IGF2BP1 may be a new target treatment of melanoma to overcome drug resistance. IGF2BP1 can promote the overexpression of several carcinogenic proteins by binding and stabilizing its mRNA. BTYNB, a new IGF2BP1 inhibitor, can selectively and effectively inhibit IGF2BP1 binding to c-myc mRNA. Small molecule inhibitors can affect the binding of RBP and mRNA to affect the stability of mRNA, which may provide a new way for the development of cancer drugs. Meanwhile, IGF2BP1 was closely related to the regulation of the cargo of EVs, which had the potential to become a new therapy related to RBP targeted therapy. In addition, some reports described the interaction between RBPs and miRNAs/lncRNAs, where RBPs interfere with miRNA/lncRNAs function, thus highlighting a new pattern of post-transcriptional regulation of gene expression (Kedde and Agami, 2008; Ferrè et al., 2016). It has been reported that IGF2BP1 interfered with the function of miR-183, influencing the stabilization of βTrCP1 mRNA in 293T cells (Elcheva et al., 2009). IGF2BP1 stabilized MITF mRNA and increased its expression and its transcriptional activity in melanoma, which was mediated by counteracting the miR-340–mediated degradation of MITF mRNA (Goswami et al., 2015). LncRNA THOR can regulate the mRNA stabilization activities of IGF2BP1 *via* binding to IGF2BP1. It is speculated that the combination of lncRNAs/miRNAs and RBP to regulate mRNA stability/gene expression provides a new idea for tumor treatment, and the development of small molecule inhibitors for RBP offers new possibilities for cancer therapy.
